# Reconstruction of microtia with crus helicis transversal deformity using flap technique combined with ear cartilage transplantation: a retrospective study

**DOI:** 10.1186/s40902-026-00510-2

**Published:** 2026-04-11

**Authors:** Yiwen Deng, Hongli Zhao, Jianguo Chen, Zexin Zhang, Peixu Wang, Senmao Wang, Wenkang Luan, Xiaobo Yu, Lin Lin, Bo Pan, Haiyue Jiang

**Affiliations:** 1 Plastic Surgery Hospital, Chinese Academy of Medical Sciences & Peking Union Medical College, Beijing, China; 2 Department of Plastic and Aesthetic (Burn) Surgery, Second Xiangya Hospital of Central South University, Changsha, China

**Keywords:** Microtia, Crus helicis transversal deformity, Ear deformity, Ear reconstruction

## Abstract

**Background:**

The combination of crus helicis transverse deformity and microtia presents one of the most complex ear deformities. Historically, the surgical correction of this deformity has predominantly utilized rib cartilage for ear reconstruction. This study presents a method for correcting microtia with crus helicis transversal deformity, utilizing the flap technique in conjunction with cartilage transplantation.

**Methods:**

From February 2017 to October 2023, a total of 42 patients with crus helicis transversal deformity and microtia were included in this retrospective study. The corrective surgery combines flap technique with cartilage transplantation. We collected the morphological data of ear, Visual Analogue Scale (VAS) satisfaction scores, and Aesthetic Outcomes Scale (AOS) aesthetic ratings of patients both prior to and 12 months following surgery.

**Results:**

The average duration of follow-up was 13.71 ± 2.97 months. Following the operation, there has been a significant improvement in the length, width, and circumference of the ear, as well as in the differences in these measurements between the two ears compared to prior to the procedure. The average AOS score prior to surgery was 17.90 ± 1.41, while the average AOS score following surgery increased to 29.57 ± 1.51. The preoperative VAS satisfaction score was recorded at 1.95 ± 1.12, while the postoperative VAS score showed a significant increase to 8.23 ± 0.87.

**Conclusion:**

The flap technique, combined with cartilage grafting, effectively corrects the crus helicis transversal deformity with microtia. The approach maximizes the use of deformity ear tissue while minimizing damage. Additionally, it creates a natural and aesthetically pleasing ear shape, resulting in high patient satisfaction.

**Level of evidence statement:**

IV.

**Supplementary Information:**

The online version contains supplementary material available at 10.1186/s40902-026-00510-2.

## Introduction

Microtia is a worldwide congenital malformation, exhibiting an overall incidence of approximately 2.06 cases per 10,000 live births [1]. The findings of recent studies on the psychological aspects in children with microtia suggest that these individuals often face significant psychological challenges arising from the appearance of ear deformity [2]. The correction of microtia not only enhances the aesthetic appearance of the patient but also fosters psychological well-being, rendering it a highly valuable surgical intervention for addressing this deformity [3,4]. The classification of microtia encompasses four distinct grades, namely grade I, grade II, grade III, and grade IV [5–7]. For grade I and II microtia, the correction technique can be applied in accordance with the methods used for ear deformity correction, without necessitating ear reconstruction [8–10]. However, microtia is frequently accompanied by anomalies in various ear subunits, thereby significantly complicating the corrective procedures for microtia.

The crus helix is a significant anatomical structure of the ear, situated in the anterior superior region of the auricle [11]. The crus helix represents a continuation of the helix, diverging from its superior margin and extending vertically towards the region above the tragus. And, the crus helix typically appears slender and gradually tapers, contributing to the distinctive curved contour of the ear and playing a pivotal role in establishing three-dimensional structure [12]. Furthermore, both the shape and symmetry of the crus helix serve as critical criteria for assessing the aesthetic quality of the ear. And the anatomical configuration of the crus helix represents a crucial region for intricate surgical interventions in auricular reconstruction and ear deformity correction.

Crus helicis transversal deformity is a congenital anomaly of the ear characterized by an abnormal protrusion of the crus helicis. This condition manifests as an excessive horizontal extension or protrusion of the crus helicis, thereby disrupting the normal anatomical structure and aesthetic appearance of the ear. The presence of crus helicis transversal deformity results in a distortion of the ear’s shape and proportion, frequently accompanied by anomalies in adjacent structures such as the helix and antihelix. Transversal deformity of the crus helicis results in an asymmetrical ear morphology, and addressing this deformity continues to pose a significant challenge in the field of auricular reconstruction.

The combination of crus helicis transversal deformity and microtia represents a relatively uncommon ear malformation that complicates the surgical correction of auricular deformities. However, there are no documented techniques available for the correction of crus helicis transversal deformity in conjunction with microtia. The present study introduces and assesses the implementation of combined flap techniques in conjunction with cartilage grafting for the correction of both crus helicis transversal deformity and microtia.

## Methods

A retrospective study was performed on patients diagnosed with unilateral microtia and crus helicis transverse deformity, who were admitted to Plastic Surgery Hospital, Chinese Academy of Medical Science & Peking Union Medical College between February 2017 and October 2023.Inclusion criteria:1.The patient presents with a distinct congenital microtia accompanied by the crus helicis transversal deformity;2.Utilize the flap technique in conjunction with cartilage grafting to correct ear deformities;3.Patients and their guardians can actively engage in surgical treatment and research initiatives. Exclusion criteria: Patients with incomplete follow-up and incomplete clinical data. A thorough retrospective analysis of the patient’s medical records and photographs was performed. The clinical data, surgical methodologies, postoperative assessments, complications encountered, and patient satisfaction were systematically evaluated. This research received approval from the ethics committee of Plastic Surgery Hospital of Peking Union Medical College(2024 − 381) and secured informed consent from both patients and their guardians. This study strictly followed the ethical principles of the Declaration of Helsinki. In addition, data presentation and statistical analysis were conducted in compliance with the STROBE (Strengthening the Reporting of Observational Studies in Epidemiology) guidelines, which are intended to improve the transparency and integrity of observational study reporting.

### Surgical procedures

Prior to the surgical procedure, the same surgeon meticulously records the patient’s ear dimensions including width, length, and circumference, while also capturing a photographic image of the affected ear. Following the successful administration of anesthesia, the surgical incision was meticulously outlined with methylene blue in accordance with the specific ear deformity. Administer a local anesthetic (0.5% lidocaine and 1/100,000 epinephrine) along the pre-marked surgical incision.

**Fig. 1:**
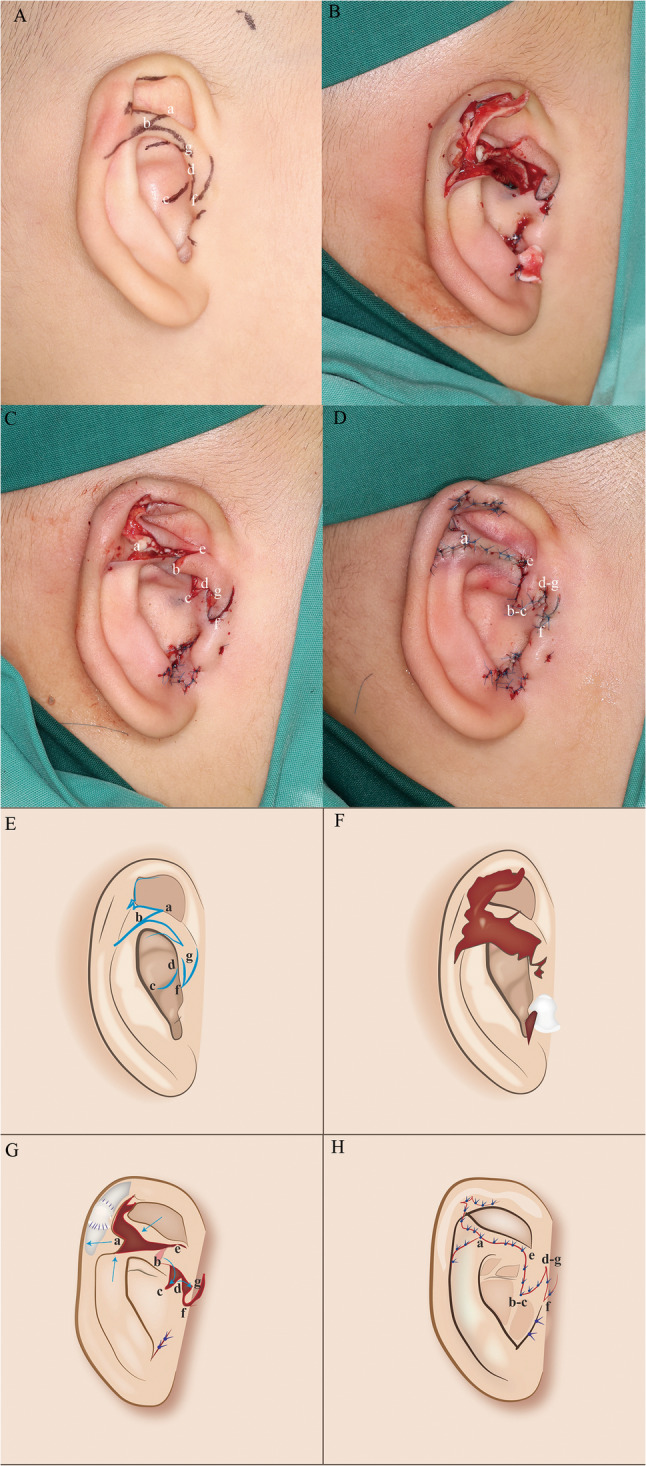
The surgical procedure for correcting crus helicis transversal deformity and congenital microtia using flap technique and cartilage grafting. **A**, **E** Preoperative surgical incision design; **B**, **F** Enlarge the intertragic notch, harvest excess cartilage from the intertragic notch and the crus helicis transversal deformity. Cut the skin along the pre-operative designed incision, and advance the flaps to correct the ear deformity; **C**, **G** Transfer cartilage to the helix area to widen the ear, and transfer flaps to cover the wound; **D**, **H** Postoperative immediate photographs; a, b, c, d, e, f, g : The apex of each flap. The flap transfer process involves the transition of point b to point c (b-c), the shift of point d to point g (d-g), and the outward displacement of point a

Correction of crus helicis transversal deformity: In the anterior region of the crus helicis transversal deformity tissue, a triangular flap was meticulously designed to align with the natural anatomical trajectory of the crus helicis, followed by a local flap transfer employed for reconstructing the crus helicis.In the posterior part of the crus helicis transversal deformity tissue, two triangular flaps were designed and used to reconstruct the superior crus helicis and the inferior crus helicis. The skin tissue surrounding the transversal deformity of the crus helicis was designed to form several flaps, aiming to enhance the concha. And the cartilage from the crus helicis transversal deformity tissue was harvested for subsequent correction of microtia (Fig. 1).

**Fig. 2:**
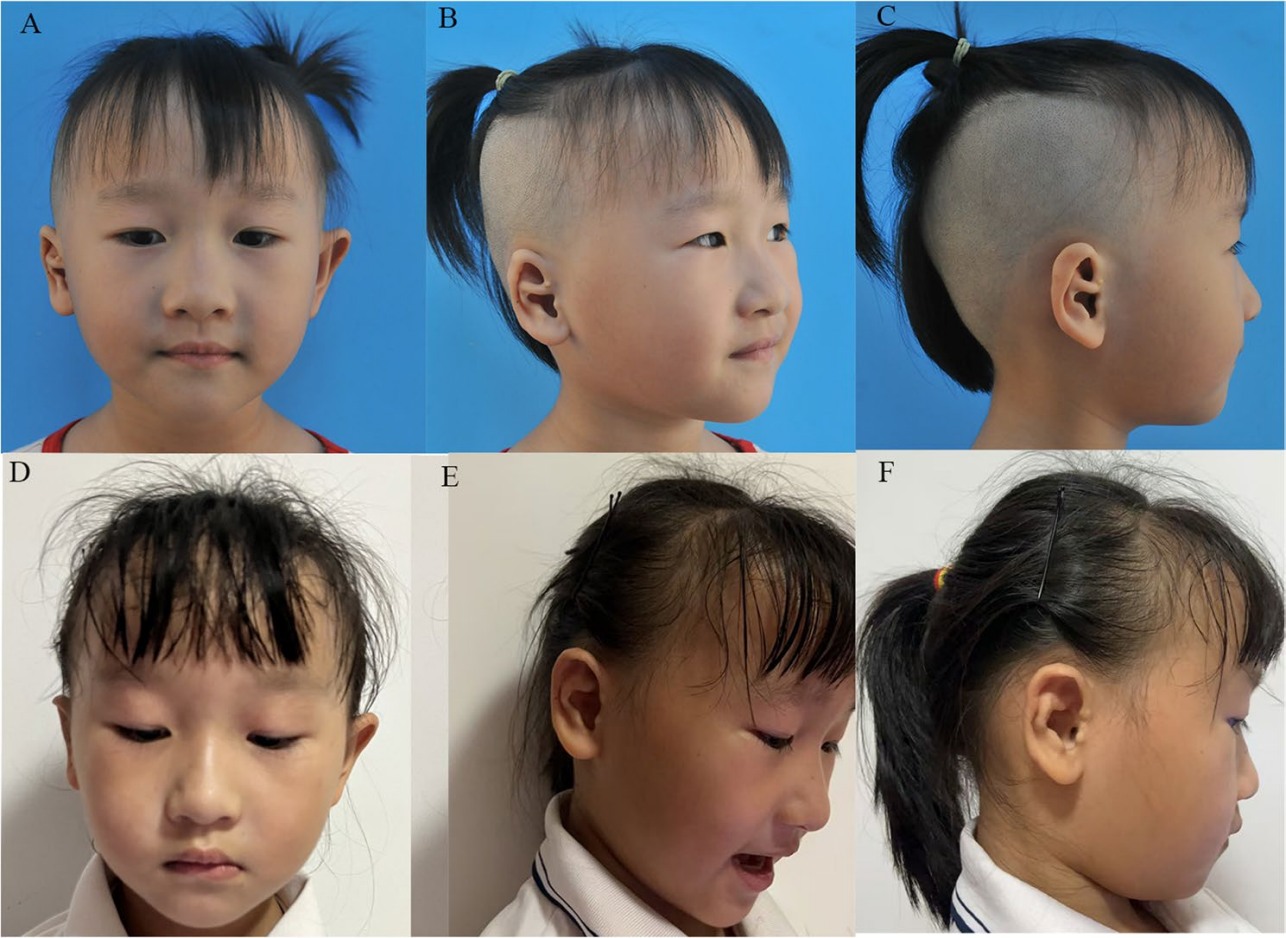
A 6-year-old patient with crus helicis transversal deformity and congenital microtia was treated with a flap combined with cartilage grafting for correction. **A**-**C** Preoperative photographs; **D**-**F** Postoperative 18 months photographs

**Fig. 3:**
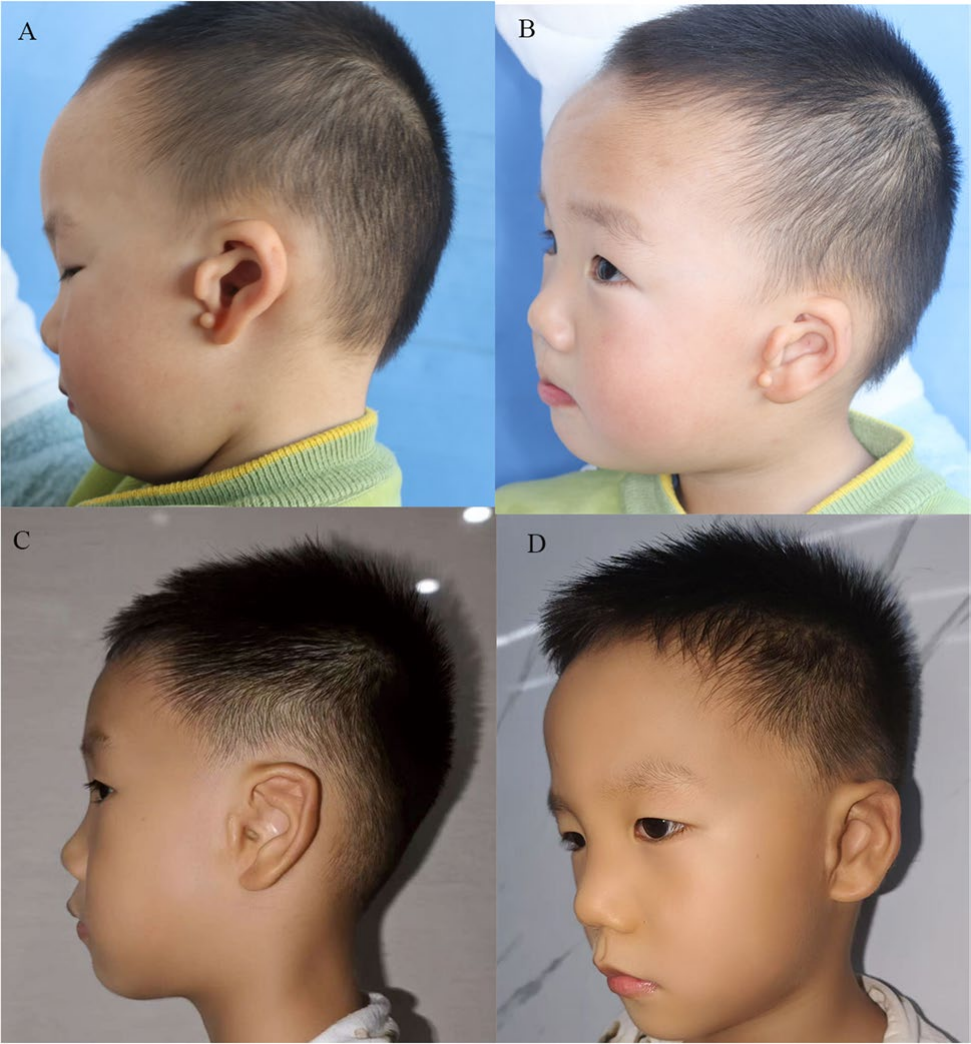
A 1-year-old patient with crus helicis transversal deformity and congenital microtia was treated with a flap combined with cartilage grafting for correction. **A**-**B** Preoperative photographs; **C**-**D** Postoperative 50 months photographs

Correction of microtia: A triangular flap was meticulously designed at the interauricular notch of the external auditory meatus, thereby enlarging the opening of the external auditory meatus and establishing the inter-auricular notch. Subsequently, the excess cartilage from the external auditory meatus was surgically harvested and preserved for potential applications in the correction of microtia. A vertical incision is performed at the apex of the helix, and horizontally elongate both ends of the cartilage to enlarge the size of the ear. Subsequently, cartilage graft harvested from the crus helicis and from the intertragic notch was transplanted into the defect site, where it was secured with absorbable sutures to restore the contour of the helix (Figs. 2 and 3 and Supplementary Material 1 ).

### Postoperative measurement

Auricle morphology data were measured using precise vernier calipers before and after surgery. The measured parameters included ear length, ear width, ear circumference, and their corresponding disparities (ear length disparity, ear width disparity, and ear circumference disparity), which were calculated by subtracting the measurement of one side from that of the other. Specific measurement landmarks were clearly defined to ensure standardization: ear length was measured from the top of the auricle to the earlobe, ear width at the widest part of the auricle, and ear circumference along the entire edge of the auricle. To ensure measurement accuracy, each parameter was measured twice by two independent doctors who did not participate in the study’s surgical procedures.Both doctors were strictly blinded to the study grouping and surgical timing to avoid measurement bias, and the final value for each parameter was taken as the average of the two measurements obtained by the two doctors.

### Evaluation of postoperative aesthetic outcomes

During the evaluation process of aesthetic outcomes, the same independent senior plastic surgeon (who did not participate in any surgical procedures of this study) meticulously scrutinized the standardized preoperative and postoperative 12-month photographs of each patient using the Aesthetic Outcomes Scale (AOS). The surgeon was strictly blinded to the timing of the photographs (preoperative vs. postoperative), and standardized photographing protocols were strictly followed to ensure consistency in shooting angle and lighting, thereby reducing assessment bias caused by inconsistent image quality [13]. To assess the aesthetic results, a Likert scale with four points (1 = poor, 2 = fair, 3 = good, and 4 = excellent) was utilized. The aesthetic evaluation criteria encompassed ear subunit structures, including the helix, crus helix, scaphoid fossa, antihelix, triangular fossa, and auriculocephalic sulcus. Additionally, the symmetry, size, shape, and incision concealment were taken into consideration.

### Patient-reported outcomes

Satisfaction with auricular morphology was evaluated by the patients’ guardian using the Visual Analogue Scale (VAS) both preoperatively and at the 12-month postoperative follow-up [14].All assessments were completed by the patients’ guardians under the guidance of the same independent physician who was not involved in the surgical procedure.A standardized terminology was adopted for the VAS survey, with scores ranging from 0 to 10; higher scores indicated greater satisfaction with the surgical outcome.

### Statistical analyses

The statistical analysis was conducted using SPSS25.0 software to input and analyze the data. The preoperative and postoperative VAS scores and AOS scores were reported as mean±standard deviation (SD) values. The paired t test was utilized to compare the scores of the subjects before and after surgery. The level of significance for statistical analysis was set at *p* < 0.05.

## Results

### Demographic information

A total of 46 patients diagnosed with microtia and transversal crus helicis deformity were initially enrolled in this study, all of whom were scheduled to undergo flap surgery combined with auricular cartilage transplantation for the correction of auricular deformities. However, 4 patients were excluded from the final data analysis due to failure to complete the postoperative follow-up, resulting in 42 patients being included in the final study cohort. The average follow-up duration was 13.71 ± 2.97 months (ranging from 12 to 24 months), with 30 cases observed on the right side and 12 cases on the left side. The study included 28 male patients and 14 female patients, with an average age of 2.92 ± 1.42 years (Table 1). The circumference and width of the ear post-surgery are significantly greater than those measured prior to the procedure. The morphology and positioning of the crus helicis have been successfully restored to their original state. The helix undergoes a noticeable widening and enlargement, accompanied by an expansion of the cavum conchae. Furthermore, there is an increased prominence in the triangular fossa.Among the enrolled patients, one case developed hypertrophic scarring during the 3-month follow-up after surgery. Intralesional injection of triamcinolone acetonide was promptly administered in the early stage, and satisfactory overall outcomes were achieved in the subsequent follow-up visits.

**Table 1:** Patient Demographics

Characteristic	Value (%)
No. of patients	42(100%)
Mean age at surgery ± SD, yr	2.92 ± 1.42
Gender	
Male	28(66.67%)
Female	14(33.33%)
Side of deformity	
Left	12(28.57%)
Right	30(71.43%)
Surgical techniques	
Local flap	42(100%)
Cartilage transplantation	42(100%)
Z-Plasty techniques	42(100%)
Dilatation of intertraginal notch	20(47.62%)
Duration of residency, day	2.02 ± 0.64
Mean operating time, hour	0.95 ± 0.15
Follow-up period, mo	13.71 ± 2.97
Ethnicity	
Asian	42 (100%)

### The auricle morphology data

The intraclass correlation coefficients (ICC) for inter-rater reliability were 0.940, 0.908, and 0.873 for preoperative auricular length, width, and circumference; 0.860, 0.885, and 0.927 for the corresponding postoperative measurements; and 0.908, 0.880, and 0.929 for the contralateral normal auricles. All ICC values indicated excellent measurement consistency.The average length of the ear prior to surgery was measured at 4.28 ± 0.33 cm, with a width of 2.38 ± 0.25 cm and a circumference of 7.56 ± 0.47 cm. In contrast, the average measurements for the opposite ear were found to be 5.31 ± 0.31 cm in length, 2.95 ± 0.25 cm in width, and a circumference of 9.26 ± 0.46 cm. Following the operation, the average length of the corrected ear measured 4.93 ± 0.31 cm, with a width of 2.76 ± 0.25 cm and a circumference of 8.80 ± 0.44 cm. Prior to surgery, the length of both ears exhibited a difference of 1.02 ± 0.19 cm, while the width showed a difference of 0.57 ± 0.12 cm, and the circumference had a difference of 1.69 ± 0.27 cm; following surgery, the differences in ear length reduced to 0.37 ± 0.13 cm, with width differences decreasing to 0.19 ± 0.08 cm, and the difference in circumference was reduced to 0.46 ± 0.21 cm. The measurements of ear length, width, and circumference, along with the differences in these dimensions between the two sides, showed significant improvement after surgery compared to preoperative values (*P* < 0.05) (Table 2).

**Table 2:** The results of auricle morphology data, visual analogue scale (*N* = 42)

	Preoperative	Postoperative	t	*P*-value
Ear length	4.28 ± 0.33	4.93 ± 0.31	-9.21	< 0.001
Ear width	2.38 ± 0.25	2.76 ± 0.25	-6.80	< 0.001
Ear circumference	7.56 ± 0.47	8.80 ± 0.44	-12.26	< 0.001
Ear length disparity	1.02 ± 0.19	0.37 ± 0.13	17.42	< 0.001
Ear width disparity	0.57 ± 0.12	0.19 ± 0.08	16.28	< 0.001
Ear circumference disparity	1.69 ± 0.27	0.46 ± 0.21	22.61	< 0.001
VAS	1.95 ± 1.12	8.23 ± 0.87	-28.54	< 0.001

The t test was employed for statistical analysis. *VAS* Visual Analogue Scale, VAS scores ranged from 0 to 10

### The results of aesthetic evaluation

The preoperative average AOS score was 17.90 ± 1.41, whereas the postoperative average AOS score significantly increased to 29.57 ± 1.51. This observed difference was statistically significant (*P* < 0.05) (Table 1). Significant improvements were observed in the helix (1.35 ± 0.48 vs. 3.47 ± 0.50), anti-helix (0.64 ± 0.48 vs. 3.04 ± 0.43), triangular fossa (0.23 ± 0.43 vs. 3.09 ± 0.61), incisura (2.35 ± 0.79 vs. 3.11 ± 0.50), and concha (1.38 ± 0.49 vs. 2.95 ± 0.62) between the pre-operative and post-operative stages (Table 3).

**Table 3:** The results of visual analogue scale and auricle aesthetic outcomes(*N* = 42)

	Preoperative score	Postoperative score	t	*P*-value
Helix	1.35 ± 0.48	3.47 ± 0.50	-19.60	< 0.001
Anti-helix	0.64 ± 0.48	3.04 ± 0.43	-23.82	< 0.001
Triangular fossa	0.23 ± 0.43	3.09 ± 0.61	-24.59	< 0.001
Tragus	3.71 ± 0.45	3.76 ± 0.43	-0.49	0.625
Incisura	2.35 ± 0.79	3.11 ± 0.50	-5.26	< 0.001
Scapha	0.88 ± 0.45	2.73 ± 0.49	-17.90	< 0.001
Concha	1.38 ± 0.49	2.95 ± 0.62	-12.83	< 0.001
Lobe	3.71 ± 0.45	3.73 ± 0.44	-0.24	0.810
Anti-tragus	3.61 ± 0.49	3.64 ± 0.48	-0.22	0.824
Total	17.90 ± 1.41	29.57 ± 1.51	-36.50	< 0.001

The t test was employed for statistical analysis. *VAS* Visual Analogue Scale; Total, Total score for each subunit of the auricle; The auricle subunits aesthetic outcomes scores ranged from 0 to 4. The total score for each subunit of the auricle ranged from 0 to 36

### The outcomes of satisfaction assessment

The preoperative VAS satisfaction score was recorded at 1.95 ± 1.12, whereas the postoperative VAS score showed a significant increase to 8.23 ± 0.87. This observed difference was statistically significant (*P* < 0.05) (Table 2). All patients’s guardians expressed satisfaction with the outcomes of the surgical procedures.

## Discussion

The intricate anatomy of the crus helicis and the difficulties associated with rectifying crus helicis deformities have consistently posed significant challenges in surgical interventions for ear deformity correction. Kiyoshi et al.^11^ proposed the utilization of preauricular appendage tissue in conjunction with flap techniques to address congenital helix and crus helicis deformities. This approach optimally leverages the preauricular appendage tissue for reconstructive purposes, ensuring adequate skin coverage for the correction of ear deformities. Yavuz [15] found the combination of V-Y advancement of the crus helicis and perichondrioplasty technique can achieve a satisfactory outcome in creating the antihelical fold. The correction of the crus helicis necessitates the comprehensive application of flap techniques for structural reshaping, and the surgical procedure must be coordinated with adjacent ear structures to ensure optimal outcomes.

Crus helicis transversal deformity is a condition where the normally subtle ridge of the antihelix becomes excessively prominent, causing aesthetic concerns or discomfort. This deformity can be classified as part of congenital ear anomalies and is often associated with misshaped cartilage in the upper ear, leading to an overly folded or angular appearance. The correction of this deformity often involves otoplasty techniques that reshape the cartilage to create a smoother and more natural contour. The presence of crus helicis transversal deformity in conjunction with microtia undoubtedly complicates the surgical procedure. Historically, the management of crus helicis transversal deformity associated with congenital microtia has predominantly utilized autologous rib cartilage for auricular reconstruction [16,17]. The harvesting of costal cartilage may lead to complications such as chest wall deformities, pain, pneumothorax, and the reconstructed ear often appears less natural than a normal ear [18]. The correction method presented in this paper optimally utilizes the residual auricular tissue while mitigating the secondary damage associated with employing costal cartilage for ear reconstruction.

The core surgical design concept aims to correct crus helicis transversal deformity via cartilage reshaping and flap rotation, thereby restoring the auricle’s natural anatomical morphology. Initially, the Z-plasty is meticulously planned during the correction of ear deformities, serving to strategically reorganize the skin and soft tissue in order to restore the ear to its normal anatomical subunit [17,19]. A series of Z-shaped incisions are meticulously crafted in the auricular region, with careful consideration given to both the orientation and length of these incisions to ensure a balanced movement of the flap in conjunction with tissue tension [20]. Moreover, the surgical procedure employs cartilage grafts harvested from the crus helicis transversal deformity to reconstruct microtia, representing an optimal utilization of tissue while minimizing additional trauma associated with procuring cartilage from alternative anatomical sites [13].Simultaneously, the depth of the concha is enhanced by rotating the flap in the area affected by crus helicis transversal deformity, thereby improving both the functional and aesthetic aspects of the ear [21]. The surgical procedure should ensure that the direction and angle of flap movement align with the natural anatomical structure of the ear, while avoiding excessive tension that could lead to necrosis of the flap. Microtia is typically associated with a narrow intertragic notch, which can be augmented during surgical procedures through the utilization of a triangular skin flap [22]. Concurrently, a portion of the cartilage within the intertragic notch may be harvested and processed as a cartilage graft to enhance the size of the helix.

Auricular reconstruction using costal cartilage also serves as a viable approach for correcting crus helicis transversal deformity. Firstly, when this reconstruction is performed with a relatively abundant supply of autologous ear cartilage, a substantial portion of the cartilage often remains underutilized. Although the reconstructed ear may present with more distinct morphological characteristics and clearer subunit definition, it tends to have a rigid texture and a somewhat unnatural appearance. Additionally, costal cartilage-based auricular reconstruction is subject to specific age-related eligibility criteria, which may limit its application in younger patients.Therefore, the selection of a surgical strategy necessitates a comprehensive weighing of multiple factors, aiming to correct crus helicis transversal deformity in a manner that optimally aligns with the clinical needs of patients as well as the aesthetic expectations of their guardians.

Our clinical studies indicate that crus helicis transversal deformity can coexist with microtia, and utilizing the tissue from the crus helicis transversal deformity can serve as a valuable source for correcting microtia. The findings of our previous research have demonstrated that the growth characteristics of ear cartilage persist even after the correction of ear deformities [23]. Therefore, we advocate early correction of ear deformities, not only to preserve normal auricular development but also to mitigate the psychological problems associated with such deformities [24]. However, this method also has certain limitations. Firstly, the surgical method mentioned in this paper is only suitable for mild microtia with transversal crus helicis deformity. For such mild deformities, the size and contour of the auricle can be effectively improved by localized adjustment of the auricular skin and cartilage. In contrast, patients with severe microtia typically require auricular reconstruction using autologous costal cartilage to achieve satisfactory correction. Moreover, this technique involves the utilization of multiple flaps, and the potential for postoperative hypertrophic scarring may impact the aesthetic appearance of the ear. In addition, microtia with transverse crus helicis deformity is frequently accompanied by cartilaginous tissue deficiency. Consequently, in the absence of alternative supporting materials, the anatomical reconstruction of auricular subunits may be suboptimal. Nevertheless, the overall auricular contour is satisfactory, and most patients along with their families are content with the correction results, thus not requesting further surgical adjustments to the auricular subunits. If further correction of the auricular subunits is indicated, costal cartilage can be harvested during the second-stage surgery to reconstruct ear subunits. Moving forward, our future research will focus on refining the technique to achieve a smooth, natural helix contour and enable the precise reconstruction of the superior crus.

## Conclusion

The crus helicis transverse deformity frequently coexists with mild microtia. By utilizing the normal anatomical structure of the ear as a reference, the combination of flap techniques and cartilage grafting can yield favorable outcomes in the correction of crus helicis transversal deformity. The tissue harvested from the transverse deformity of the crus helicis can be utilized as a viable source for the correction of microtia. The surgical technique described herein is characterized by its ease of operation, brief treatment duration, minimal trauma to patients, and high level of patient satisfaction, rendering it a valuable reference for clinical practice.

## Supplementary Information

Supplementary Material 1.

## Data Availability

No datasets were generated or analysed during the current study.
