# Supplementary figures and images for: Reconstruction of microtia with crus helicis transversal deformity using flap technique combined with ear cartilage transplantation: a retrospective study
Source: Maxillofac Plast Reconstr Surg. 2026 Apr 11;48(1):9. doi: 10.1186/s40902-026-00510-2 (PMC13129075; doi:10.1186/s40902-026-00510-2)

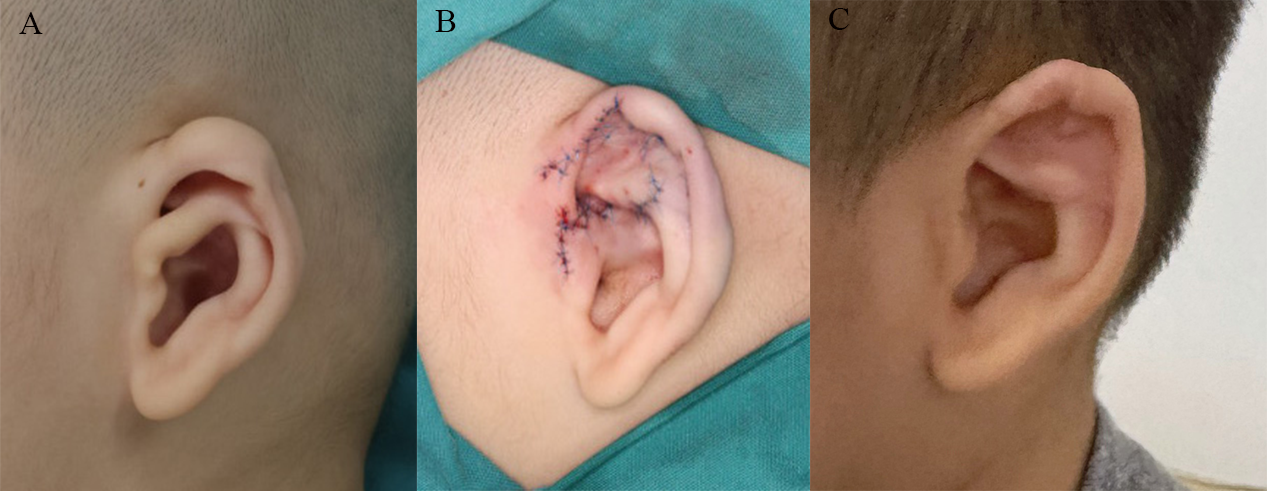

Supplement: Supplementary file 1 — Supplementary Material 1. [file 40902_2026_510_MOESM1_ESM.png]
